# Photosynthetic activity and metabolic profiling of bread wheat cultivars contrasting in drought tolerance

**DOI:** 10.3389/fpls.2023.1123080

**Published:** 2023-02-02

**Authors:** Abdul Ghaffar, Nadeem Hussain, Rahaf Ajaj, Suzan Marwan Shahin, Hussan Bano, Muhammad Javed, Ayesha Khalid, Memoona Yasmin, Kausar Hussain Shah, Muhammad Zaheer, Muhammad Iqbal, Zafar Ullah Zafar, Habib-ur-Rehman Athar

**Affiliations:** ^1^ Institute of Botany, Bahauddin Zakariya University, Multan, Pakistan; ^2^ Department of Environmental and Public Health, College of Health Sciences, Abu Dhabi University, Abu Dhabi, United Arab Emirates; ^3^ College of Arts and Science, Umm Al Quwain University, Umm Al Quwain, United Arab Emirates; ^4^ Department of Botany, The Women University, Multan, Pakistan; ^5^ Department of Botany, University of Education, Lahore, Pakistan; ^6^ Department of Botany, University of Okara, Okara, Pakistan

**Keywords:** drought stress, mineral nutrients, osmotic adjustment, *Triticum aestivum*, chlorophyll fluorescence, water relations

## Abstract

The rapid increase in population growth under changing climatic conditions causes drought stress, threatening world food security. The identification of physiological and biochemical traits acting as yield-limiting factors in diverse germplasm is pre-requisite for genetic improvement under water-deficit conditions. The major aim of the present study was the identification of drought-tolerant wheat cultivars with a novel source of drought tolerance from local wheat germplasm. The study was conducted to screen 40 local wheat cultivars against drought stress at different growth stages. Barani-83, Blue Silver, Pak-81, and Pasban-90 containing shoot and root fresh weight >60% of control and shoot and root dry weight >80% and 70% of control, respectively, P (% of control >80 in shoot and >88 in root), K^+^ (>85% of control), and quantum yield of PSII > 90% of control under polyethylene glycol (PEG)-induced drought stress at seedling stage can be considered as tolerant, while more reduction in these parameters make FSD-08, Lasani-08, Punjab-96, and Sahar-06 as drought-sensitive cultivars. FSD-08 and Lasani-08 could not maintain growth and yield due to protoplasmic dehydration, decreased turgidity, cell enlargement, and cell division due to drought treatment at adult growth stage. Stability of leaf chlorophyll content (<20% decrease) reflects photosynthetic efficiency of tolerant cultivars, while ~30 µmol/g fwt concentration of proline, 100%–200% increase in free amino acids, and ~50% increase in accumulation of soluble sugars were associated with maintaining leaf water status by osmotic adjustment. Raw OJIP chlorophyll fluorescence curves revealed a decrease in fluorescence at O, J, I, and P steps in sensitive genotypes FSD-08 and Lasani-08, showing greater damage to photosynthetic machinery and greater decrease in JIP test parameters, performance index (PI_ABS_), maximum quantum yield (Fv/Fm) associated with increase in Vj, absorption (ABS/RC), and dissipation per reaction center (DIo/RC) while a decrease in electron transport per reaction center (ETo/RC). During the present study, differential modifications in morpho-physiological, biochemical, and photosynthetic attributes that alleviate the damaging effects of drought stress in locally grown wheat cultivars were analyzed. Selected tolerant cultivars could be explored in various breeding programs to produce new wheat genotypes with adaptive traits to withstand water stress.

## 1 Introduction

Global wheat production is forecast at 783.8 million tons in 2022 (FAOSTAT, 2022). Global human population is expected to exceed 9 billion by 2050, requiring at least 60% increase in wheat yield (Sekher, 2022). The drought puts 22 million people at risk of starvation by September 2022, and it triggered global economic losses about 124 billion US $ from 1998 to 2017, according to a report from the United Nations World Food Programme (WFP and UNICEF, 2022). The percentage of vegetation affected by drought has more than doubled in the last 40 years, and it has affected more people worldwide than any other natural hazard. Up to 26% of the usable area of the earth is subjected to drought and approximately 12 million hectares (Mha) of land is lost each year due to drought worldwide (FAO et al., 2022). Out of the total 79.6 Mha land of Pakistan, 62 Mha is vulnerable to drought stress while about 27% of land under cultivation is facing drought stress (FAOSTAT, 2022). Drought resistance in crop plants includes avoidance and tolerance. In recent years, different strategies have been adopted to minimize the challenges associated with drought, such as supplemental irrigation, modern dry land farming, mulching, artificial precipitation, ground water recharge, and use of compatible and drought-tolerant genotypes. Breeding is one of the most efficient options to overcome drought stress through the development of new genotypes adapted to drought (Blum, 2016). Different morpho-physiological traits of wheat that are hampered under drought stress include plant height, leaf area, chlorophyll content, stomatal conductance, relative water content, osmotic potential, water potential, and photosynthesis, so the mechanism of damage can be understood by studying these attributes under drought stress (Ahmad et al., 2022; Chen et al., 2022a; Peršić et al., 2022). Reduced uptake of N, P, and K^+^ was observed in plants under drought stress. Low N uptake under dry conditions affects leaf water relations, chlorophyll fluorescence, and other photosynthetic attributes restricting growth and yield (Sallam et al., 2019). Drought stress increases concentration of soluble sugars, proline, and anti-oxidant enzymes activities to combat reactive oxygen species (ROS) (Tefera et al., 2021). Reduction in protein content under drought stress may be due to protein hydrolysis or oxidative inhibition of protein synthesis (Ozturk et al., 2021). However, Halder et al. (2022) described that drought stress caused the accumulation of osmoprotectant proteins (dehydrins) in drought-tolerant wheat cultivars to protect plant cellular structures from drought-induced damages. These proteins are highly hydrophilic and impart drought stress tolerance by enhancing the water retention capacity, elevating chlorophyll content, maintaining photosynthetic machinery, activating ROS detoxification, and promoting the accumulation of compatible solutes (Riyazuddin et al., 2022)

Plants cannot utilize sufficient light energy under drought stress, so electron transport chain is disturbed, leading to the production of ROS, which damage thylakoid membranes (Zhu et al., 2021). The structural and functional activity of PSII can be evaluated by fast chlorophyll *a* fluorescence technique to screen drought-tolerant and drought-sensitive varieties of a particular plant species (Kalaji et al., 2018; Bashir et al., 2021; Li and Kim, 2021). Fluorescence transients from Fo (minimal fluorescence when all reaction centers are open) to Fm (maximum fluorescence when all reaction centers are closed) are called OJIP curves, which can be analyzed by JIP test and used to evaluate plant responses against drought stress (Tsimilli-Michael, 2020). The O–J phase tells about the PSII subunits connectivity and reduction in the acceptor side of PSII, J–I represents reduction in PQ pool, while the reduction in acceptor side of PSI is explained by the I–P phase (Hussain et al., 2022). Drought stress disturbs electron transfer at acceptor and donor sides of PSII, damages OEC activity, affects energetic connectivity among PSII subunits, and reduces electron transfer capacity and redox state of PSI (Zhou et al., 2019).

Plants use different mechanisms to combat drought stress such as completing life cycle before severe water stress (drought escape), conserving water by closing stomata and reducing leaf area (drought avoidance), and by osmotic adjustment (drought tolerance) (Qayyum et al., 2021). During osmotic adjustment, plants accumulate various organic and inorganic solutes (sugars, polyols, amino acids, etc.) under water-deficit conditions to maintain water status of plants and thus turgor potential to sustain photosynthesis (Zhang et al., 2022). There is little progress in developing drought-tolerant wheat cultivars due to limited knowledge about the mechanism of drought tolerance in crops, dramatic change in some physiological parameters in plants under drought stress, and genotype and environment interaction. It is suggested that information from morphological, physiological, biochemical, gene expression, genetic studies, and breeding must be integrated to understand complexity of drought tolerance in crops. Screening of available germplasm against drought stress is an important strategy in plants to cope up with the harmful effects of water deficit (Tefera et al., 2021). It is hypothesized that a novel source of drought tolerance is present in new local germplasm collection of wheat, which can be used for developing drought-tolerant wheat cultivars through selective breeding. However, pre-breeding requires a detailed characterization of germplasm. The current study was aimed to assess genotypic variability in physiological and biochemical traits in new local germplasm collection of wheat, particularly those that are related to photosystem-II activity. The secondary objective of the study was to draw the relationship between studied traits and degree of drought tolerance. The selected cultivars having traits with high heritability and positive correlation with grain yield may be used as donors in breeding programs.

## 2 Materials and methods

### 2.1 Screening of germplasm against PEG-induced drought stress under hydroponic conditions

The study was conducted at Botanic Gardens, Bahauddin Zakariya University, Multan (30°15 N and 71°30 E). Germplasm of 40 wheat cultivars collected from Ayub Agriculture Research Institute (AARI) Faisalabad and Regional Agricultural Research Institute (RARI) Bahawalpur, Pakistan, was surface sterilized with 5% sodium hypochlorite solution for 5 min to avoid any fungal infection and washed thoroughly with distilled water. A total of 40 bowls of 24 × 24 × 10 cm (length × width × depth) size obtained from G.M. Scientific Store, Multan, Pakistan, were taken and divided into two groups of 20 bowls each (**Supplementary Figure S1**). Seeds of two varieties (10 of each) were sown in each bowl in two separate rows. One group of plants (control) was filled with Hoagland nutrient solution (Hoagland and Arnon, 1950) having 1-in. thick layer of plastic microbeads (inert polyethylene), while the treatment block was given 10% PEG-8000 solution in Hoagland solution to induce drought stress. The plants were harvested after 14 days of germination, and various parameters including shoot and root biomass (fresh and dry) and N, P, and K^+^ contents were measured.

### 2.2 Appraisal of morpho-physiological attributes of selected wheat cultivars under drought stress at adult stage

The selected drought-tolerant (Barani-83, Blue silver, Pak-81, and Pasban-90) and drought-sensitive (FSD-08, Lasani-08, Punjab-96, and Sahar-06) cultivars were further grown till adult stage, and differences in morpho-physiological and yield responses between tolerant and sensitive cultivars were explored under water stress conditions. A total of 80 plastic pots each with a diameter of 28 cm were taken. Eight seeds of each variety were sown in a pot filled with 8 kg fertile and well-textured garden soil of 18% field capacity and 36% soil saturation. Ten days after sowing, four equidistantly placed and uniformly sized plants were left in each pot after thinning. The experiment was arranged in completely randomized design (CRD) with eight cultivars, two treatments (control and drought), and five replicates. Initially, all the pots were irrigated with tap water, but after 2 weeks of germination, half of the plants were subjected to drought stress by withholding water (pre-anthesis drought cycle) till wilting state becomes evident, while the other group of plants were irrigated continuously with tap water. At this stage, different morpho-physiological and biochemical parameters were measured. Two of the four plants were harvested, and the experiment continued till the end, and finally, yield attributes were determined.

### 2.3 Measurements

#### 2.3.1 Growth attributes

Plant length was calculated by using a measuring tape from the soil surface to the tip of the longest spike. Flag leaf area was determined by the Muller (1991) formula, which is given as: flag leaf area = maximum length × maximum breadth × 0.74. Total chlorophyll content (SPAD) was estimated by using a portable chlorophyll meter SPAD 502 (Minolta, chlorophyll meter, SPAD-502, Japan) as proposed by Del Pozo et al. (2016). The middle part of the third mature leaf of each plant was used to measure the quantum yield (QY) of PSII by using handheld FluorPen (FP-100 MX-LM, Photon System Instruments, Czech Republic). A weak measuring light was used to measure Fo, and then, a saturated pulse of 3,000 µmol m^−2^ s^−1^ was applied to measure Fm. QY of PSII was calculated using the formula: QY = Fv/Fm = (Fm – Fo)/Fm.

Fresh weight of the shoot and root was noted with the help of electric balance (BSM-220.4, Hanchen, China) after separating the shoot from the root; then, plants were dried in an electric oven (UN-110, Memmert, Germany) at 75°C for 72 h, and dry biomass was noted.

#### 2.3.2 Leaf water relations

The second leaf from the top was cut from the main tiller to measure the water potential of the leaf (Ψw) at early in the morning using a Scholander-type pressure chamber (Arimad-2, Japan) as described by Scholander et al. (1964). The same leaf was kept in a freezer at −20°C for a week and thawed by a glass rod to extract cell sap, which was used for measuring osmotic potential (Ψs) by an osmo-meter (Vapro, 5520, USA). Turgor potential (Ψp) was calculated from the difference in Ψw and Ψs by the formula as described by Nobel (1991), i.e., Ψp = Ψw − Ψs. Fresh leaf samples were weighed (Fw) and dipped in water in the dark for 24 h so that they become turgid, and their turgid weight (Tw) was noted. These leaves were then dried in an oven at 80°C for 48 h, and the dried weight (Dw) was noted. The formula that was used to determine relative water content as described by Jones and Turner (1978) is RWC (%) = [(Fw − Tw)/(Fw − Dw)] × 100.

#### 2.3.3 Organic osmolytes

Fresh leaf sample (0.25 g) was ground in 3% sulfo-salicylic acid (5 ml) in a chilled pestle and mortar. The homogenate was filtered, and 2 ml was taken in a test tube in which 2 ml acid ninhydrin solution (1.25 g ninhydrin+20 ml of 6M orthophosphric acid+30 ml glacial acetic acid) and 2 ml glacial acetic acid were added and heated in a boiling water bath for 1 h at 100°C, and the reaction was terminated by cooling in ice bath. Four milliliters of toluene was added in the test tube, vortexed for 15–20 s, and allowed to stand, and then, the upper layer (chromophore) was taken. Absorbance was recorded at 520 nm using a spectrophotometer, and the concentration of proline was determined with the help of a standard curve (Bates et al., 1973). Proteins were calculated using bovine serum albumin (BSA) calibration curve on fresh weight basis (Bradford, 1976), while the concentration of total free amino acids was measured following the Hamilton et al. (1943) protocol. Total soluble sugars were estimated by standard methods as proposed by Yemm and Willis (1954). In 0.2 g dried ground leaf sample taken in a test tube, 10 ml of 80% ethyl alcohol was added and shaken overnight. The supernatant was taken in plastic bottles after three to four times washing with water and then made the volume of each sample up to 50 ml with distilled water. A 0.3 ml extract was put in a test tube and 3 ml of anthrone solution added and heated for 10 min in a water bath and cooled, and absorbance was recorded at 625 nm by a UV spectrophotometer.

#### 2.3.4 Mineral nutrients and malondialdehyde

The digestion mixture (Se, 0.42g; Li_2_SO_4_.2H_2_O_2_, 14g dissolved in 350 ml of H_2_O_2_ and 420 ml conc. H_2_SO_4_) was used for plant tissue digestion. A total of 100 mg of dried ground plant material was digested in 2 ml of digestion mixture, adding HClO_4_ (0.5 ml) on a hot plate. The digested mixture was diluted to 50 ml with distilled water and filtered, and K^+^ was measured by a flame photometer (Jenway, PFP-7) following the method of Allen et al. (1985). P contents were determined by a spectrophotometer (Jenway, 6850, UK), measuring absorption at 470 nm (Jackson, 1958). Leaf N contents were estimated according to Kjeldhal method (Bremner, 1965). Leaf lipid peroxidation can be estimated by measuring MDA content, adopting thCakmak and Horst (1991) method.

#### 2.3.5 Yield-related parameters

The number of tillers per plant, number of spikelet per spike, and number of grains per spike were counted; spike length was measured by a measuring tape, while 100 grain weight and grain yield per plant were measured by using electric balance.

#### 2.3.6 Fast chlorophyll *a* fluorescence transient (OJIP)

Chlorophyll *a* fluorescence was measured from the middle part of the third mature leaf of each plant (40 min after sunset to ensure dark adaptation of leaves and then exposed to strong actinic light of 3,000 μmol m^−2^ s^−1^), with a handheld chlorophyll fluorescence meter (FluorPen FP-100 MX-LM, Photon System Instruments, Czech Republic) from 20 μs to 2 s. The data were recorded based on literature available on websites of chlorophyll fluorescence meter manufacturers using computer software FlourPen v 1.0.4.0. According to Strasser et al. (2004), the JIP test parameters used to measure energy distribution, flux ratios, and performance index include ABS/RC, TRo/RC, ETo/RC, and DIo/RC (absorption, trapped energy, electron transport, and dissipation energy flux per reaction center) and Fo, Fj, Fi, and Fm (minimum fluorescence, fluorescence intensity at J phase, fluorescence intensity at I phase, and maximum fluorescence). Fv, Vj, and Vi represent variable fluorescence, relative variable fluorescence at phase J, and relative variable fluorescence at phase I of the fluorescence transient curve. Fm/Fo = electron transport rate through PSII, Fv/Fo = ratio of photochemical to non-photochemical quantum efficiency, Fv/Fm = maximum quantum yield of primary photochemistry, while PI_ABS_ is the photosynthetic performance index on absorption basis. ΦPSII shows the effective quantum yield of PSII photochemistry, ΦDo explains maximum quantum yield of non-photochemical de-excitation, ΦEo represents quantum yield of electron transport, and ψo = ETo/TRo explains the yield of electron transport per trapped excitation.

### 2.4 Statistical analysis

The adult experimental plan was a two-factor factorial completely randomized design with eight cultivars, two treatments, and five replications. Data were analyzed by two-way analysis of variance (ANOVA) using the computer program CoStat (Version 6.303, USA). Means ± SE were compared with least significant difference (LSD) test at 5% level of significance by Snedecor and Cochran (1989). Graphical representation of data was performed by MS Excel software.

## 3 Results

### 3.1 Screening and selection of local wheat germplasm against drought stress at seedling stage

**Figure 1:**
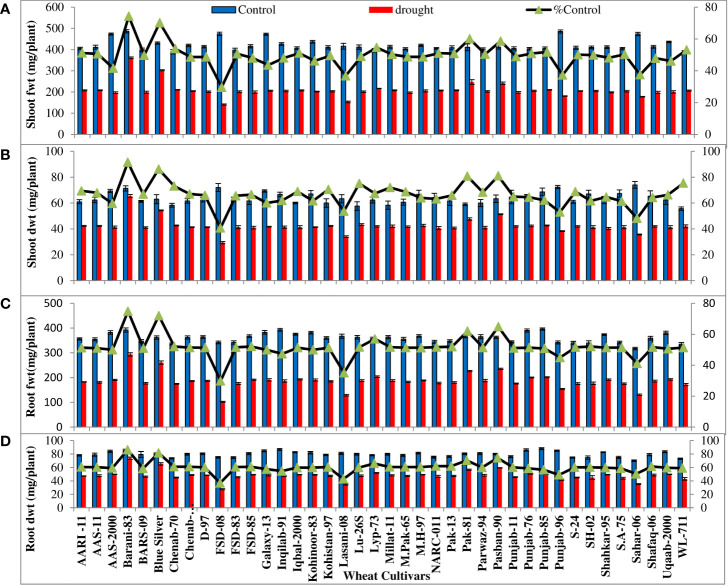
Shoot and root fresh and dry weights (mg/plant) of wheat seedlings, when plants of 40 local wheat cultivars were grown under normal or PEG-induced drought stress for 2 weeks. Means (± SE; n=5) are presented on the primary vertical axis, while % of the control values are presented on the secondary vertical axis. **(A)** Shoot fwt, **(B)** shoot dwt, **(C)** root fwt, and **(D)** root dwt.

**Figure 2:**
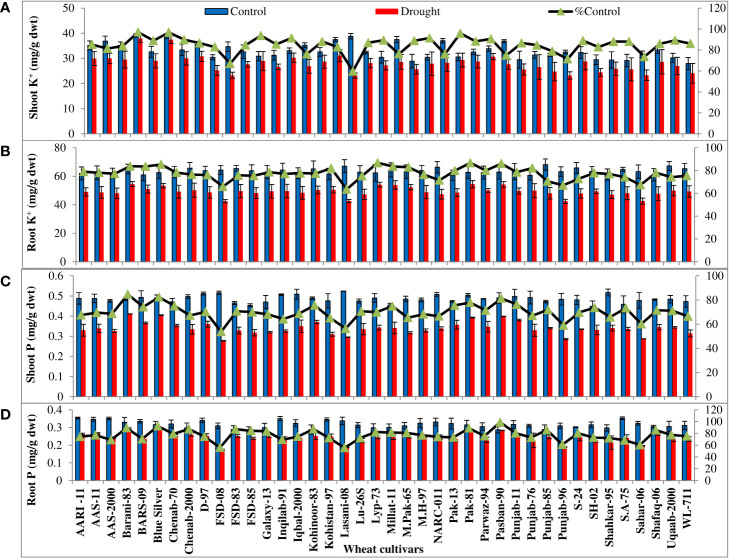
Shoot and root K^+^ and P accumulation in wheat seedlings when plants of 40 local wheat cultivars were grown under normal or PEG-induced drought stress for 2 weeks. Means (± SE; n=5) are presented on the primary vertical axis, while percent of the control values is presented on the secondary vertical axis. **(A)** Shoot K^+^, **(B)** root K^+^, **(C)** shoot P, and **(D)** root P.

All the wheat cultivars showed significant (p ≤ 0.001) reduction in shoot and root fresh and dry biomass and K**^+^** and P accumulation under water-deficit conditions. The extent of reduction in biomass and K^+^ and P accumulation in the leaves and roots varied considerably in all wheat cultivars. The percentages of control values of shoot and root fresh and dry weight were maximum in four cultivars (Barani-83, Blue silver, Pak-81, and Pasban-90), while FSD-08, Lasani-08, Punjab-96, and Sahar-06 showed minimum percent of control values of shoot and root fresh and dry weight due to PEG-induced water stress (**Figures 1A-D**). Shoot and root K^+^ concentration calculated on dry weight basis decreased under drought stress. Maximum percent of control or minimum decrease was observed in cultivar Barani-83 (97% of control of shoot and 83% of control of root), followed by Blue Silver, Pak-13, Pak-81, and Pasban-90. However, a maximum decrease or minimum percent of control was observed in FSD-08 (67% of control of the shoot and 65% of control of the root) followed by Lasani-08, Punjab-96, and Sahar-06 under drought stress conditions (**Figures 2A, B**). Shoot and root P concentration decreased in all the cultivars under drought stress. A maximum decrease was observed in FSD-08 (54% of control of shoot and 56% of control of root) and then come varieties Lasani-08 (56% of control of the shoot and root) followed by Punjab-96 and Sahar-06 under drought stress conditions. A minimum decrease was observed in Barani-83 with 85% and 89% of control of the shoot and root, respectively, followed by Blue Silver, Pak-81, and Pasban-90 under water scarce conditions (**Figures 2C, D**).

### 3.2 Assessment of differential morphological and physio-biochemical responses of selected wheat cultivars under drought stress

#### 3.2.1 Shoot and root fresh and dry biomass, plant height, flag leaf area, quantum yield of PSII, and total chlorophyll content (SPAD)

**Figure 3:**
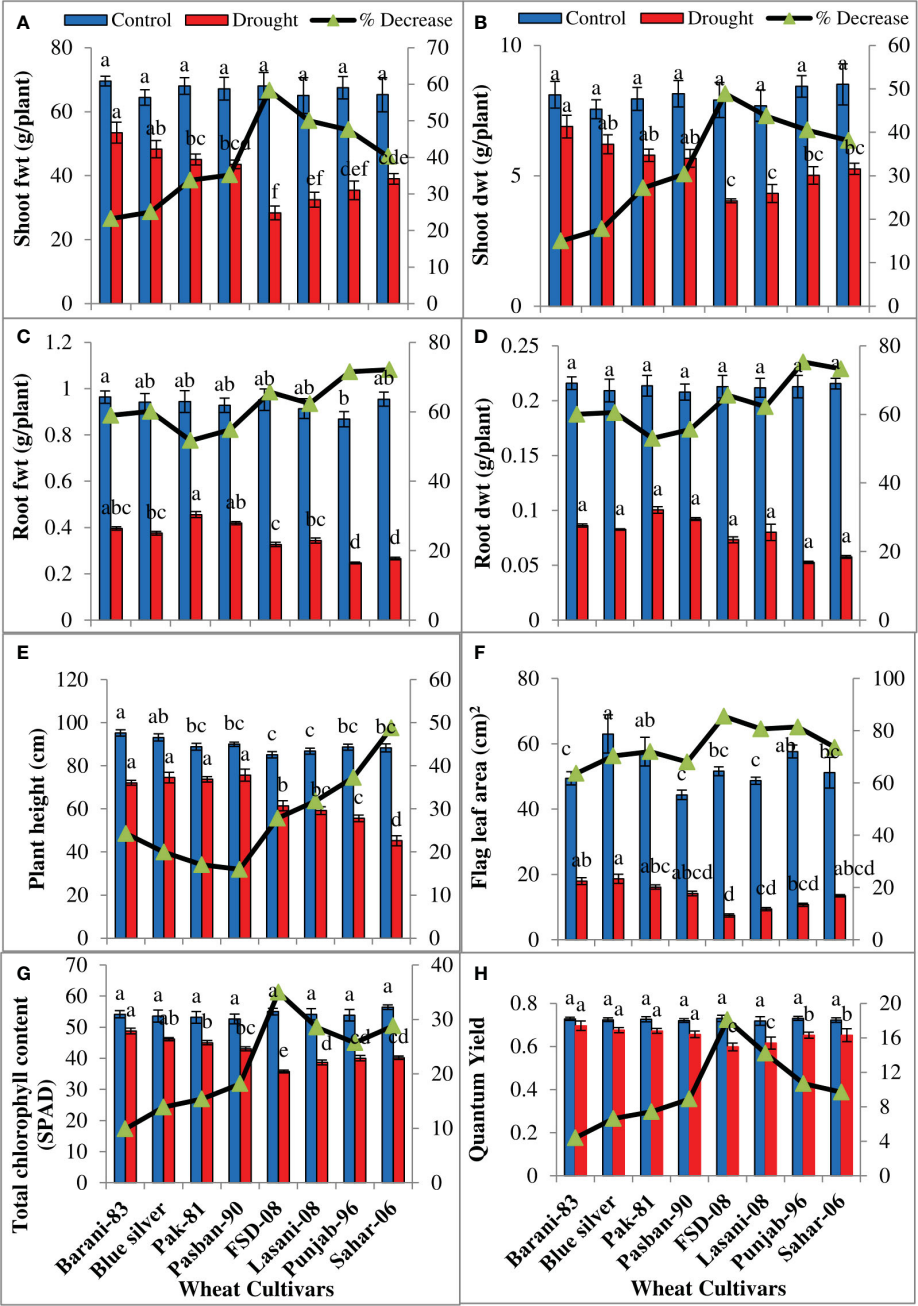
Growth attributes of selected wheat cultivars subjected to control or drought stress at adult stage. Means (± SE; n=5) are presented on the primary vertical axis, while percent decrease value is presented on the secondary vertical axis. Different letters represent significant differences among cultivars at p < 0.05 according to LSD test. **(A)** Shoot fresh weight, **(B)** shoot dry weight, **(C)** root fresh weight, **(D)** root dry weight, **(E)** plant height, **(F)** flag leaf area, **(G)** total chlorophyll content (SPAD), and **(H)** quantum yield of PSII.

Drought stress reduced shoot and root fresh and dry biomass significantly (p ≤ 0.001) in all wheat cultivars (**Table 1**) examined in the present study. Less decrease was observed in fresh and dry biomass of the shoot and root in Barani-83 than Blue Silver, Pak-81, and Pasban-90; however, maximum decrease in FSD-08 followed by Lasani-08, Punjab-96, and Sahar-06 was found under drought stress (**Figures 3A-D**). ANOVA of the data showed significant (p ≤ 0.001) decrease in plant height in all wheat cultivars under drought stress (**Table 1**). Differences among cultivars were also significant with respect to plant height; a lesser decrease (16%) in plant height in Pasban-90, while greater decrease in Pak-81 (17%), Blue Silver (20%), and Barani-83 (24%), respectively, was observed, whereas Sahar-06 showed maximum decrease (48%) in plant height, followed by Punjab-96 (37%), Lasani-08 (32%), and FSD-08 (28%), respectively, under water stress conditions (**Figure 3E**). Although the cultivars differed significantly based on flag leaf area, total chlorophyll contents, and quantum yield of PSII under drought stress (**Table 1**), the reduction in flag leaf area, total chlorophyll contents, and quantum yield of PSII was 85%, 35%, and 18%, respectively, in FSD-08; 81%, 28%, and 14%, respectively, in Lasani-08; and only 63%, 10%, and 4% in Barani-83; while 70%, 14%, and 6%, respectively, in Blue Silver was noted under the influence of water stress. The varieties Pak-81, Pasban-90, Punjab-96, and Sahar-06 show intermediate reduction in flag leaf area, total chlorophyll contents, and quantum yield under drought stress (**Figures 3F-H**).

Table 1Mean squares from ANOVA of the data for growth parameters, water relation parameters, mineral nutrients, MDA, organic osmolytes, and yield attributes of eight wheat (*Triticum aestivum* L.) cultivars subjected to drought stress at adult growth stage.

**Table:** 

Source of variation	Df	Shoot fwt	Shoot dwt	Root fwt	Root dwt		Plant height
Genotypes (G)	7	200.907***	2.552***	0.024***	6.170***		615.563***
Drought (D)	1	13,728.2***	138.811***	6.882***	0.360***		13,936.5***
G × D	7	169.643***	2.476***	0.0112***	7.654***		274.972***
Error	64	30.276	0.603	0.00224	1.247		11.168
Total	79

**Table:** 

Source of variation	Df	Flag leaf area	Chlorophyll content(SPAD)	Quantum yield of PSII	Water potential		Osmotic potential
Genotypes (G)	7	78.082*	17.859**	0.002***	0.0488***		0.0122***
Drought (D)	1	18,960.85***	2,117.5***	0.217***	18.509***		0.0107***
G × D	7	128.551***	26.298***	0.002***	0.0816***		0.0107**
Error	64	28.491	4.425	2.52e−4	0.00469		0.00273
Total	79

**Table:** 

Source of variation	Df	Turgor potential	Relative water content	Shoot N	Shoot P		Shoot K^+^
Genotypes (G)	7	0.0268***	88.928***	0.731***	0.601***		27.118***
Drought (D)	1	2.126***	2491.2***	53.203***	60.302***		1,465.01***
G × D	7	0.0182***	88.179***	0.943***	0.530***		7.2577*
Error	64	0.00321	2.0042	0.0335	0.0322		3.0043
Total	79

**Table:** 

Source of variation	Df	MDA	Total soluble sugars	Proline	Total soluble proteins		Total free amino acids
Genotypes (G)	7	6.651***	48.115***	103.742***	0.343***		40.364***
Drought (D)	1	468.262***	6,505.436***	3,430.401***	19.080***		1,967.57***
G × D	7	2.400ns	55.377***	105.0595***	0.411***		46.0643***
Error	64	1.341	8.0432	1.221	0.0218		0.900
Total	79

**Table:** 

Source of variation	Df	Grain yield/plant	100 grain weight	No. of grains/spike	No. of spikelets/spike		Spike length
Genotypes (G)	7	44.206***	0.723***	93.707***	23.771***		2.0416**
Drought (D)	1	3,614.4***	28.043***	3,380***	744.2***		533.028***
G × D	7	61.813***	0.381***	27.142ns	10.142**		0.906ns
Error	64	2.116	0.0932	17.15	3.025		0.517
Total	79

**Table:** 

Source of variation	Df	No. of tillers/plant					
Genotypes (G)	7	15.783***					
Drought (D)	1	1,015.312***					
G x D	7	3.369ns					
Error	64	1.962					
Total	79						

*, **, *** significant at 0.05, 0.01, and 0.001 probability levels, respectively.

ns, non-significant; Df, degree of freedom.

#### 3.2.2 Water relations parameters

**Figure 4:**
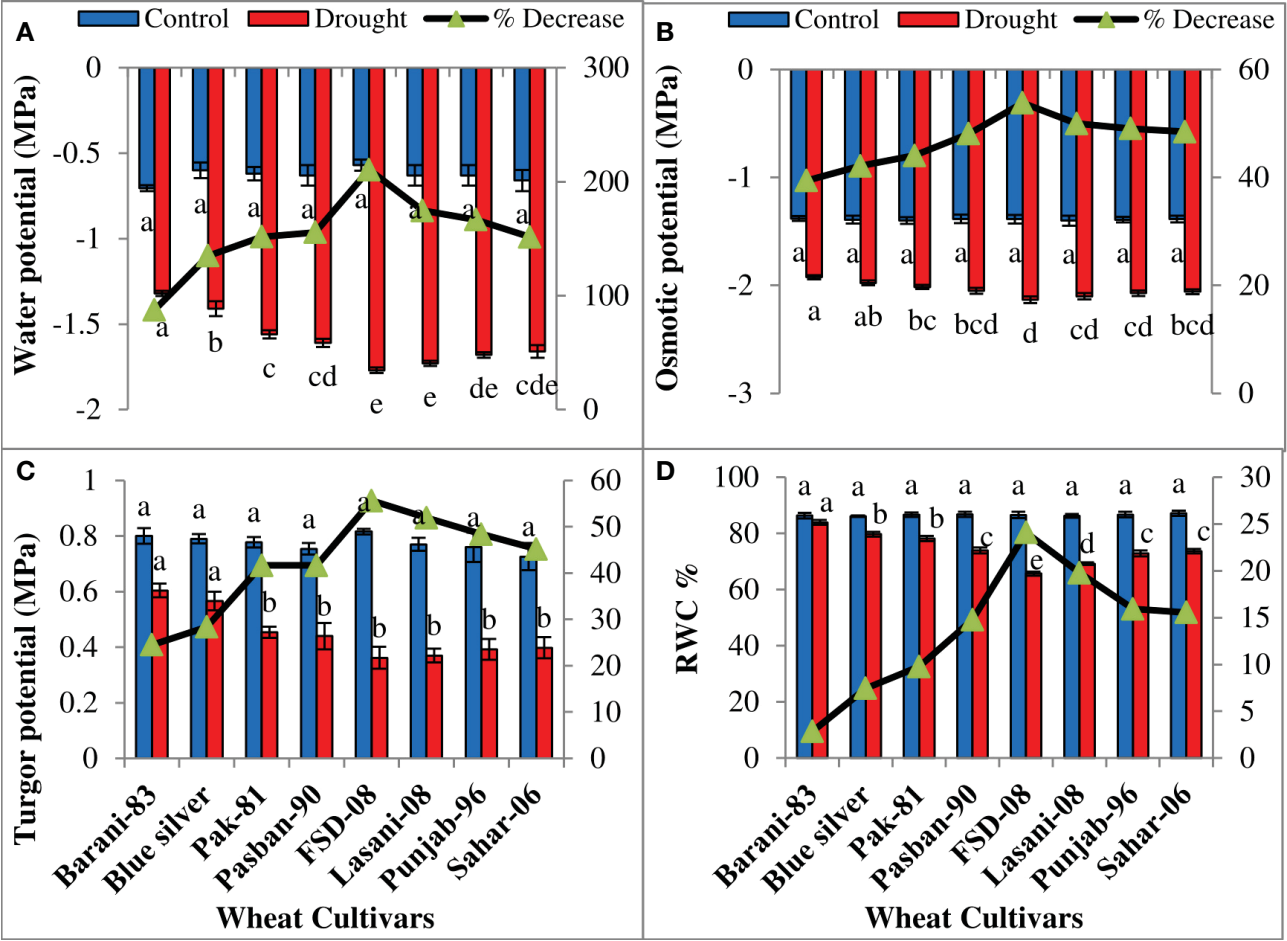
Water relations attributes of selected wheat cultivars subjected to control or drought stress at adult stage. Means (± SE; n=5) are presented on the primary vertical axis, while percent decrease value is presented on the secondary vertical axis. Different letters represent significant differences among cultivars at p < 0.05 according to LSD test. **(A)** Water potential, **(B)** osmotic potential, **(C)** turgor potential, and **(D)** % relative water content.

ANOVA of the data explained significant (p ≤ 0.001) decrease in all water relation parameters in all wheat cultivars on exposure to drought stress (**Table 1**). Drought stress reduced water potential (Ψ_w_) in all wheat cultivars; however, a minimum decrease (87%) in Barani-83 while more in Blue Silver, Pak-81, and Pasban-90 was noted due to the effect of drought stress. A maximum decline in Ψ_w_ in FSD-08 (210%) followed by Lasani-08, Punjab-96, and Sahar-06 under drought conditions was observed (**Figure 4A**). As far as osmotic potential (Ψs) is concerned, Barani-83 showed a minimum decrease (39%) followed by Blue Silver, Pak-81, and Pasban-90, which shows more decrease in the respective order; however, genotype FSD-08 showed maximum decrease (53%), followed by Lasani-08, Punjab-96, and Sahar-06, showing more decrease, respectively, under drought stress conditions (**Figure 4B**). Drought stress caused minimum reduction (24%) in turgor potential (Ψp) in Barani-83 while maximum (55%) in cv. FSD-08 (**Figure 4C**). Reduction in relative water content (RWC) due to drought stress was only 3% in Barani-83 followed by Blue Silver, Pak-81, and Pasban-90 with 7%, 10%, and 15% decrease, respectively, as compared to control. A maximum decrease (24%) in RWC was seen in FSD-08 followed by Lasani-08, Punjab-96, and Sahar-06 with 20%, 16%, and 15% decrease, respectively, due to drought stress (**Figure 4D**).

#### 3.2.3 Mineral nutrients and MDA

**Figure 5:**
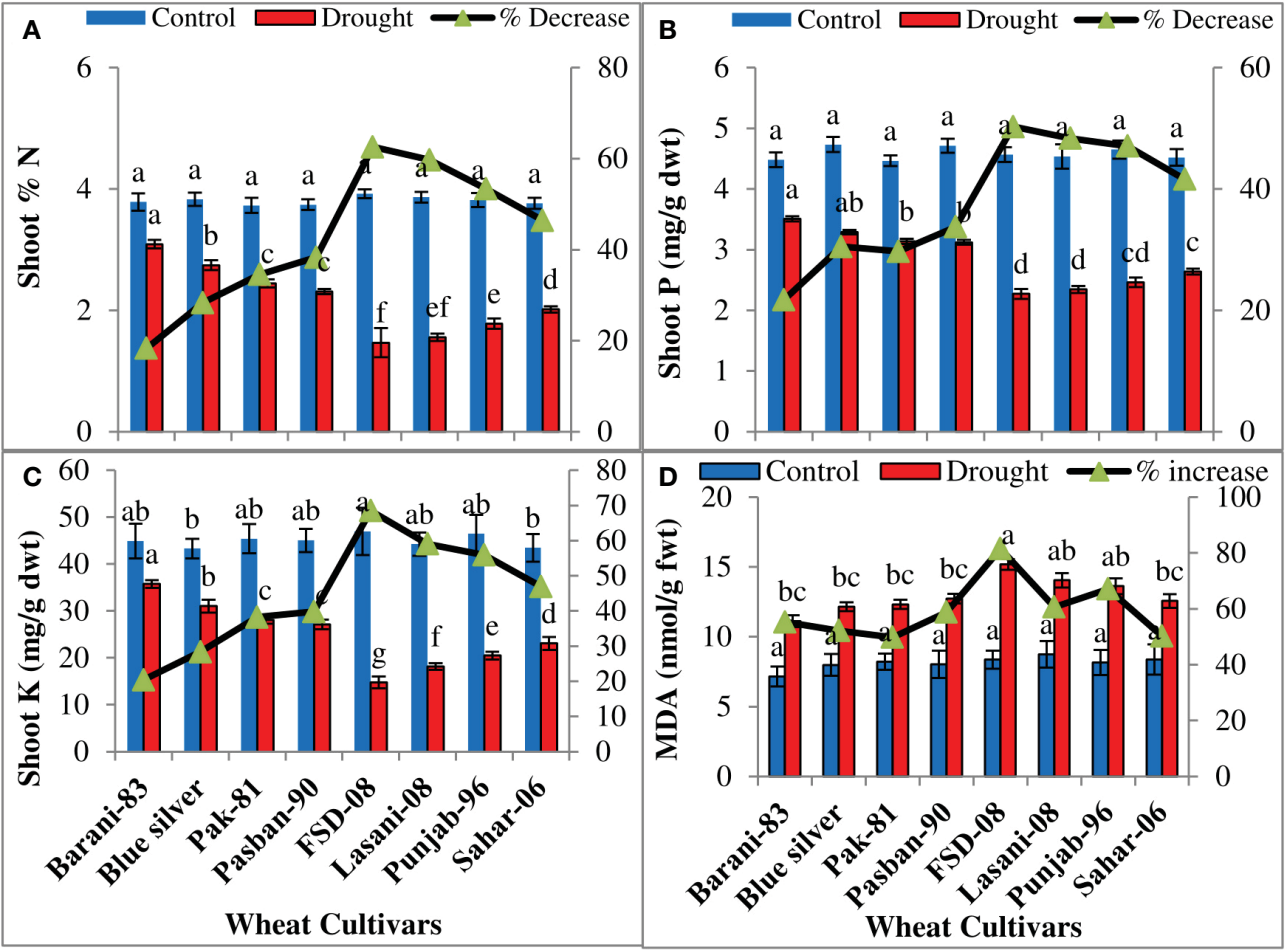
Accumulation of mineral nutrients and MDA in selected wheat cultivars subjected to control or drought stress at adult stage. Means (± SE; n=5) are presented on the primary vertical axis, while percent decrease value is presented on the secondary vertical axis. Different letters represent significant differences among cultivars at p < 0.05 according to LSD test. **(A)** Shoot N, **(B)** shoot P, **(C)** shoot K^+^, and **(D)** MDA.

The percentage of N, P, and K^+^ of the shoot was significantly (p ≤ 0.001) reduced among the wheat cultivars under drought conditions (**Table 1**). The cultivars differed significantly in accumulation of mineral nutrients in leaves; a slightly less decrease in N in Barani-83 (18%), Blue Silver (28%), Pak-81(34%), and Pasban-90 (38%) was observed, while a decreasing order of N was 62%, 59%, 53%, and 46% in FSD-08, Lasani-08, Punjab-96, and Sahar-06 cultivars, respectively (**Figure 5A**). The percentage decrease in P was least (21%) in Barani-83 followed by Blue Silver (30%), Pak-81 (29%), and Pasban-90 (33%). The cv. FSD-08 retained the least P contents with 50% decrease, while Lasani-08, Punjab-96, and Sahar-06 cultivars exhibited 48%, 47%, and 41% decrease, respectively, under drought stress conditions and may be considered as drought-sensitive cultivars (**Figure 5B**). Maximum K^+^ contents under drought conditions were retained by cv. Barani-83 and showed least reduction (20%), while Blue Silver, Pak-81, and Pasban-90 showed 28%, 38%, and 39% decrease, respectively. On the other hand, the maximum percent decrease was shown by FSD-08, i.e., 68% followed by Lasani-08, Punjab-96, and Sahar-06 varieties with 59%, 56%, and 47% decrease, respectively, due to water stress (**Figure 5C**). MDA contents represent a measure of lipid peroxidation by ROS. A significant increase in MDA concentration (p ≤ 0.001) was observed in all wheat cultivars under the influence of drought stress (**Table 1**). Drought stress caused less damage to plasma membranes in Barani-83, Blue Silver, Pak-81, and Pasban-90, i.e., less lipid peroxidation as compared to FSD-08, Lasani-08, Punjab-96, and Sahar-06 wheat cultivars, which exhibit a significant increase in MDA contents (**Figure 5D**).

#### 3.2.4 Organic osmolytes

**Figure 6:**
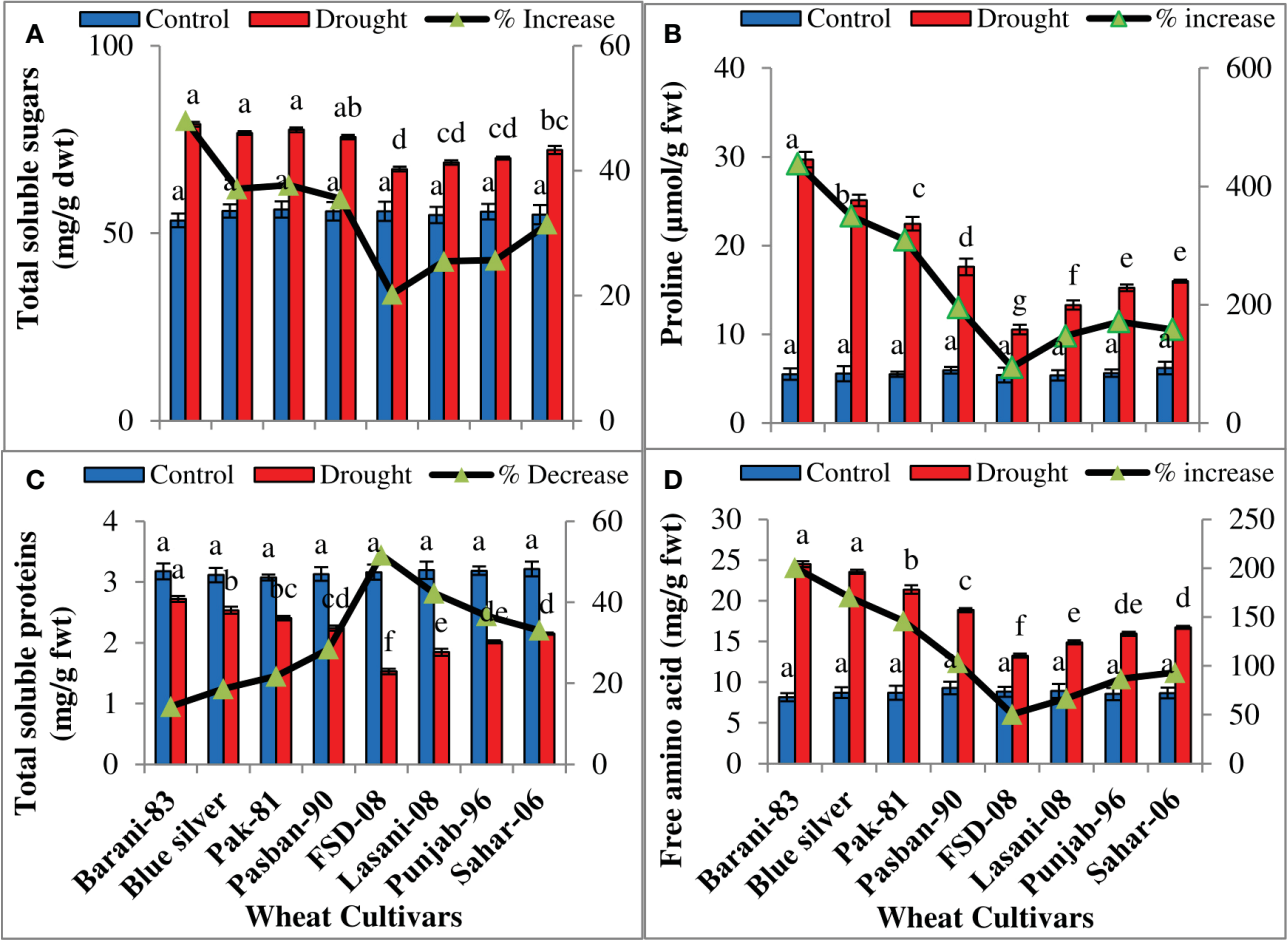
Accumulation of organic osmolytes in selected wheat cultivars subjected to control or drought stress at adult stage. Means (± SE; n=5) are presented on the primary vertical axis, while percent increase/decrease value is presented on the secondary vertical axis. Different letters represent significant differences among cultivars at p < 0.05 according to LSD test. **(A)** Total soluble sugars, **(B)** proline, **(C)** total soluble proteins, and **(D)** free amino acids.

Statistical results showed a significant (p ≤ 0.001) (**Table 1**) increase in total soluble sugars, proline, and free amino acids while a decrease in total soluble proteins in response to drought stress in all wheat cultivars. Cultivars differed considerably in accumulation of total soluble sugars under drought stress. A maximum increase (48%) in total soluble sugars was noted in cv. Barani-83 due to drought stress as compared to its control, while Blue Silver, Pak-81, and Pasban-90 with 37%, 38%, and 35% increase, respectively, come next to it. The lowest increase was displayed by FSD-08, i.e., 20%, followed by Lasani-08, Punjab-96, and Sahar-06 cultivars with ~25%–31% increase on facing drought stress (**Figure 6A**). A positive correlation was found between the degree of drought tolerance and proline accumulation. Barani-83 accumulated maximum proline and can be considered as the most drought tolerant, while FSD-08 with least increase in accumulation of proline (94%) was considered as drought sensitive (**Figure 6B**). Total soluble protein concentration diminished, while those of total free amino acids increase significantly (p ≤ 0.001) in all wheat cultivars under drought stress (**Table 1**). Barani-83, Blue Silver, Pak-81, and Pasban-90 exhibited decrease in total soluble proteins from ~14%–28%, while FSD-08, Lasani-08, Punjab-96, and Sahar-06 wheat cultivars showed greater decrease, i.e., 52%, 42%, 37%, and 33%, respectively, under dry conditions. Cultivars showing greater decrease in protein contents have greater increase in total free amino acids contents (**Figures 6C, D**).

#### 3.2.5 Yield attributes

**Figure 7:**
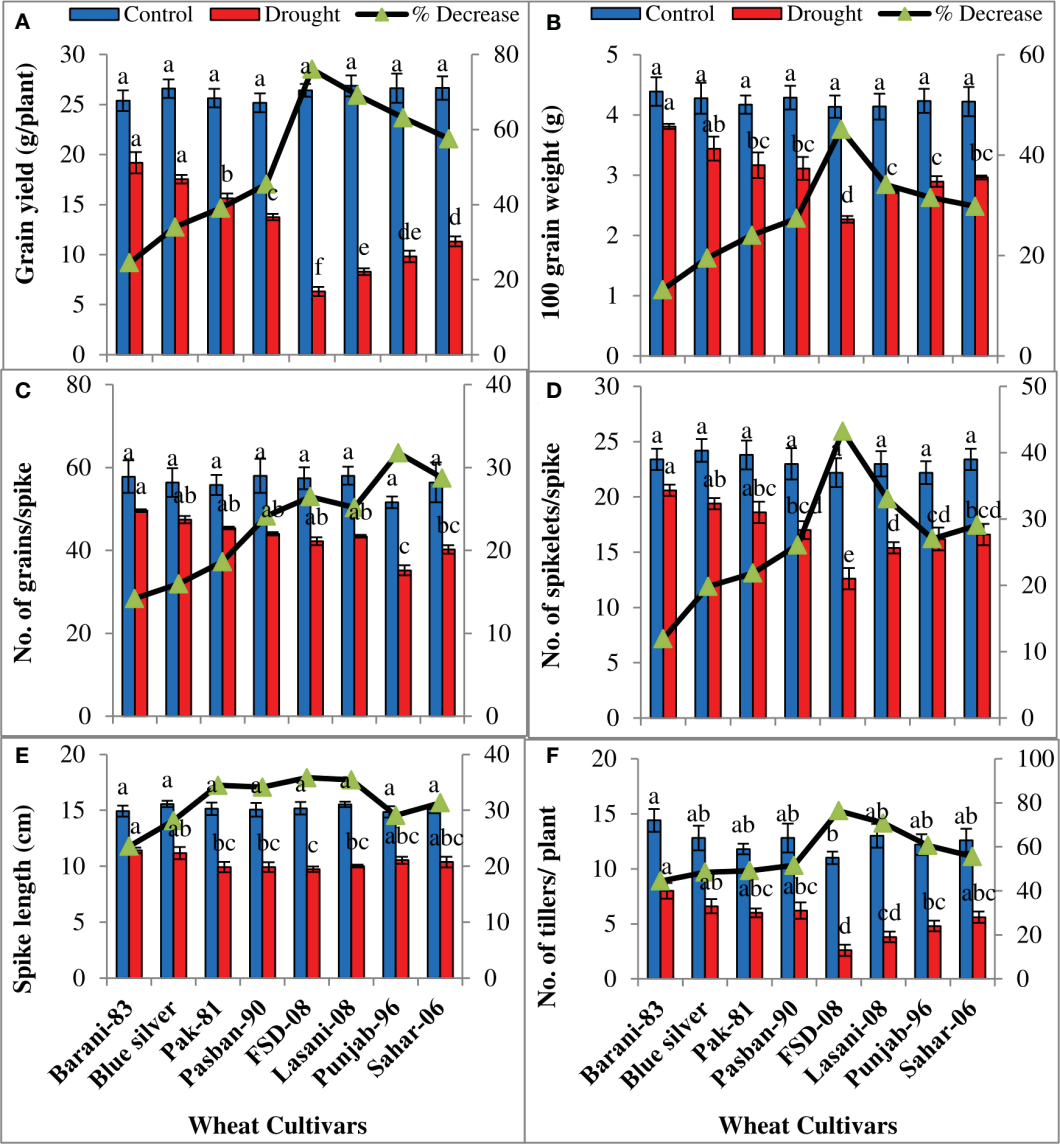
Yield attributes of selected wheat cultivars subjected to control or drought stress at adult stage. Means (± SE; n=5) are presented on the primary vertical axis, while percent decrease value is presented on secondary vertical axis. Different letters represent significant differences among cultivars at p < 0.05 according to LSD test. **(A)** Grain yield, **(B)** 100 grain weight, **(C)** number of grains/spike, **(D)** number of spikelets/spike, **(E)** spike length, and **(F)** number of tillers/plant.

Analysis of variance of the data showed significant (p ≤ 0.001) decrease in all yield parameters under water deficiency (**Table 1**). Under drought conditions, the highest grain yield (g/plant) with minimum decrease (24%) was recorded in Barani-83 followed by Blue Silver, Pak-81, and Pasban-90 with ~34%–45% decrease and may be considered as drought resistant, while the least grain yield was shown by FSD-08 with 76% decrease, followed by Lasani-08, Punjab-96, and Sahar-06 with 69%, 63%, and 57% decrease, respectively, and are thought to be drought-sensitive cultivars (**Figure 7A**). Similarly, a lesser decrease in 100-grain weight (g) was observed in Barani-83, Blue Silver, Pak-81, and Pasban-90, while a greater decrease was observed in FSD-08, i.e., 45%, followed by Lasani-08 (34%), Punjab-96 (31%), and Sahar-06 (30%) as depicted in **Figure 7B**. The number of grains per spike in Barani-83 decreased much less under drought stress followed by Blue Silver, Pak-81, and Pasban-90, however, a greater reduction was found in FSD-08 followed by Lasani-08, Punjab-96, and Sahar-06 (**Figure 7C**). The production potential of wheat is indicated by the number of spikelets per spike, which is adversely affected by drought stress in all wheat cultivars studied. Barani-83 with the least decrease (12%) in the number of spikelets per spike is followed by Blue Silver, Pak-81, and Pasban-90, whereas FSD-08 exhibited 43% decrease in the number of spikelets per spike, Lasani-08, Punjab-96, and Sahar-06 come after it (**Figure 7D**). The maximum length of spike was attained by the well-watered crop, which was substantially reduced under dry conditions. It was found that Barani-83 exhibited minimum percent reduction (23%) in spike length, while FSD-08 showed maximum percent reduction in spike length (36%) when plants faced water stress environment. The other cultivars were intermediate in reducing spike length on exposure to drought stress (**Figure 7E**). The number of fertile tillers per plant is an important indicator of plant yield, which is substantially decreased in all cultivars due to drought stress. Minimum percent reduction (44%) was shown by Barani-83 followed by Blue Silver, Pak-81, and Pasban-90 with 48%, 49%, and 51% decrease in the number of fertile tillers per plant, respectively. However, the maximum decrease (76%) was displayed by FSD-08 followed by Lasani-08, Punjab-96, and Sahar-06 with 71%, 60%, and 56% decrease, respectively, under drought stress (**Figure 7F**).

#### 3.2.6 Chlorophyll *a* fluorescence transient

**Figure 8:**
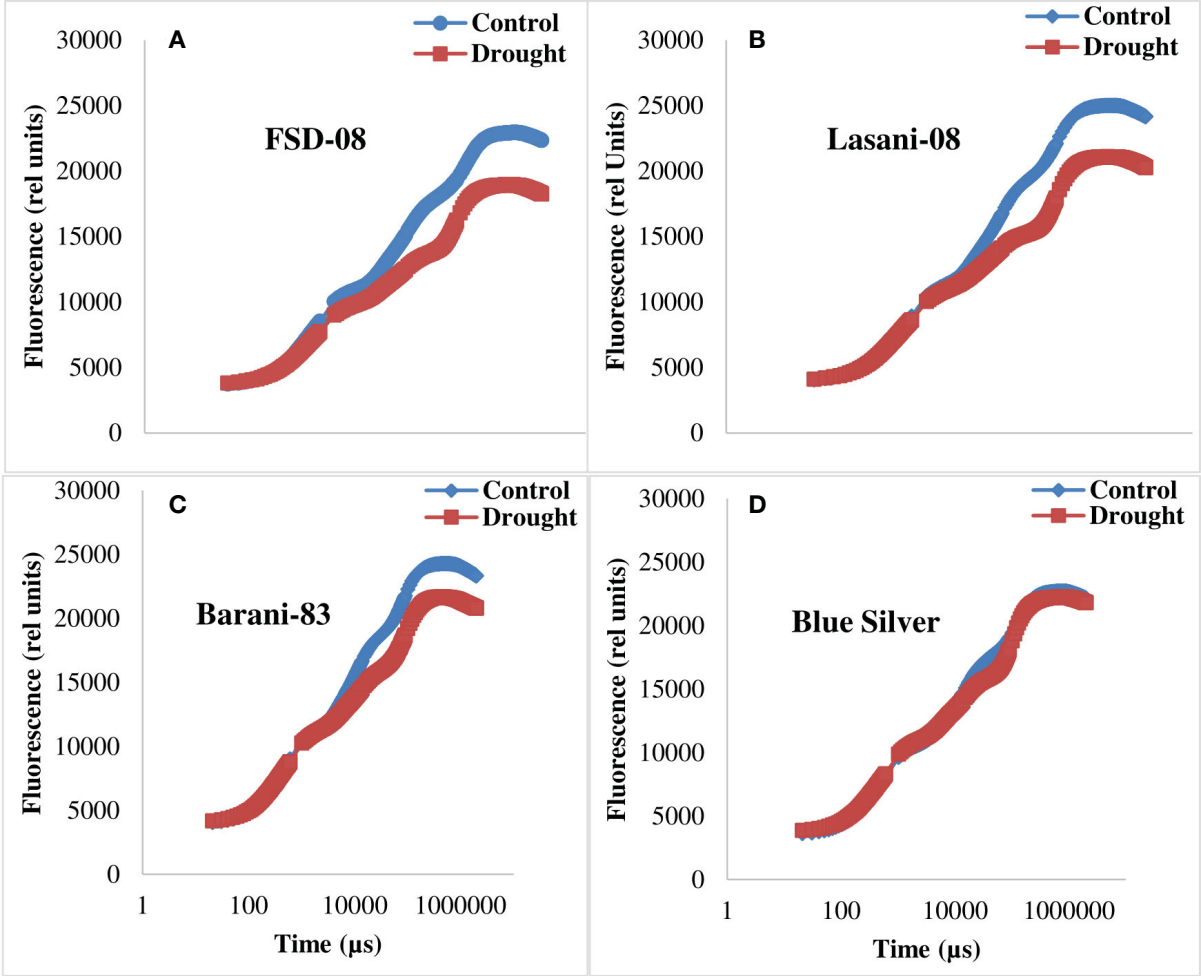
Raw OJIP chlorophyll *a* fluorescence curves (Ft) of the four selected wheat cultivars when plants were subjected to control or drought stress at adult stage. **(A)** FSD-08, **(B)** Lasani-08, **(C)** Barani-83, and **(D)** Blue Silver.

**Figure 9:**
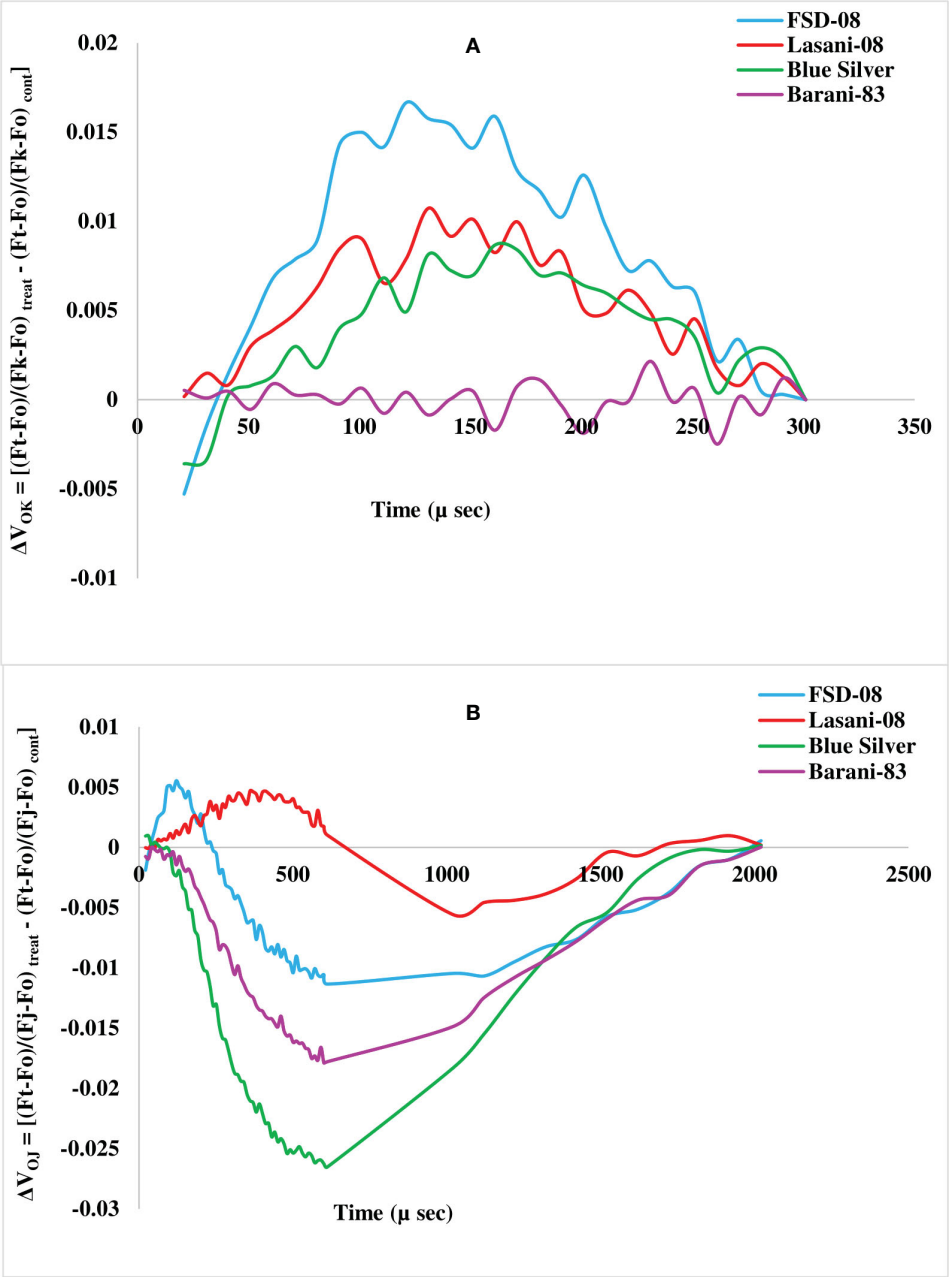
Kinetic differences of four selected wheat cultivars subjected to control or drought stress at adult stage. **(A)** Double normalized between Fo and Fk (L-band). **(B)** Double normalized between Fo and Fj (K band).

**Figure 10:**
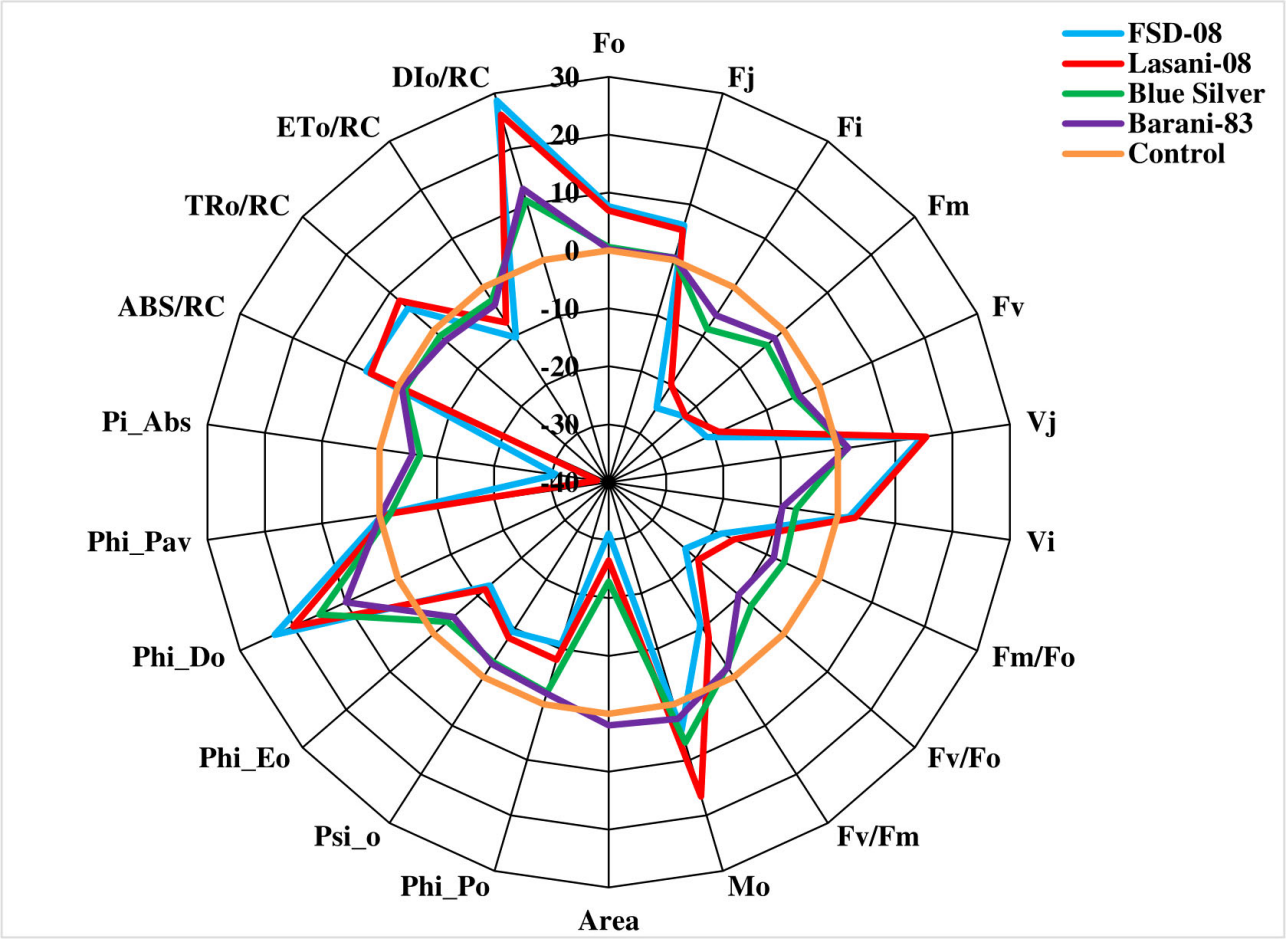
Radar plot of selected JIP test parameters showing changes in chlorophyll *a* fluorescence transients (normalized to control as reference) of four selected wheat cultivars subjected to drought stress at adult growth stage.

Chlorophyll *a* fluorescence transients (Ft) measured by JIP test explain that no considerable change in typical OJIP kinetics was observed in cv. Blue Silver, while a small decrease in chlorophyll fluorescence in cv. Barani-83 was found at I and P steps; however, in cvs. FSD-08 and Lasani-08, a greater decrease in fluorescence was observed at J, I, and P steps as clearly seen from **Figures 8A-D**. Kinetic differences between double normalized drought stressed and control plants for chlorophyll *a* fluorescence values over a time range of 20–300 μs (between O and K steps) is called ΔV_OK_ or L-band, which expresses uncoupling of OEC. Its increased value in FSD-08, Lasani-08, and Blue Silver indicated uncoupling of OEC with core protein of PSII, while in Barani-83, no such L-band was observed (**Figure 9A**). ΔV_OJ_ (K-band) represents kinetic differences between double-normalized drought stressed and control plants over a time range of 20–2,000 μs (between O and J steps) and expresses OEC activity. FSD-08 and Lasani-08 showed positive K-band values, while Blue Silver and Barani-83 showed negative values (**Figure 9B**). Fo and Fj values in cvs. FSD-08 and Lasani-08 significantly increased, whereas they remained unchanged in cvs. Blue Silver and Barani-83 under drought stress (**Figure 10**). A greater decrease in Fi, Fm, and Fv in FSD-08 and Lasani-08 as compared to Blue Silver and Barani-83 was observed under dry conditions. The relative variable chlorophyll fluorescence at J (Vj) was significantly higher in cvs. FSD-08 and Lasani-08 than that of cvs. Blue Silver and Barani-83 (**Figure 10**). The functional status of PSII is represented by various chlorophyll fluorescence ratios (Fm/Fo, Fv/Fo, and Fv/Fm). Under water scarcity, a greater decrease in all of these ratios was found in cvs. FSD-08 and Lasani-08 as compared to those of cvs. Blue Silver and Barani-83 (**Figure 10**). The quantum efficiency of primary photochemistry (ΦPo), trapped energy (Ψo), and electron transport (ΦEo) was greatly decreased in FSD-08 and Lasani-08, while a lesser decrease in these values in cvs. Blue Silver and Barani-83 was observed under water-deficient conditions (**Figure 10**). Increase in energy fluxes for absorption (ABS/RC), trapping (TRo/RC), and heat dissipation (DIo/RC) was observed in drought-stressed plants of cvs. FSD-08 and Lasani-08, whereas the energy fluxes for electron transport (ETo/RC) decreased due to imposition of drought stress (**Figure 10**). A greater decrease in PI_ABS_ (performance index of PSII) due to drought stress was observed in cvs. FSD-08 and Lasani-08 than that of cvs. Blue Silver and Barani-83 (**Figure 10**).

## 4 Discussion

Drought-induced osmotic stress at vegetative, flowering, or grain filling stage adversely affects crop production. In the present study, PEG treatment decreased shoot and root fresh and dry weight of seedlings in all wheat cultivars (**Supplementary Figure S2**) as observed previously by Peršić et al. (2022) that morpho-physiological and biochemical traits of seedlings shoot and root are greatly affected by PEG-induced drought stress reducing growth. Different growth parameters (plant height, length of roots, and fresh and dry matter weight), number of branches, and plant yield in *Cleome amblyocarp* are significantly decreased due to increasing levels of water stress (Shahin et al., 2018a). Reduction in growth attributes such as fresh and dry biomass and RWC in *Brassica napus* was observed by Chen et al. (2022b). Our results indicated that water stress led to a decline in shoot and root fresh and dry weights, with FSD-08 as the most affected and sensitive cultivar in contrast to Barani-83, the least affected and most tolerant variety under drought stress conditions (**Figures 3A-D**). Previously, it was observed that drought stress destroys photosynthetic pigments, disturbs PSII activity, and reduces stomatal conduction, influx of CO_2_, and its fixation by Calvin cycle enzymes (Ashraf and Harris, 2013). In present studies, the decrease in plant height and leaf area under drought stress (**Figures 3E, F**) might be due to protoplasmic dehydration, which in turn decreases turgidity, cell enlargement, and cell division. A reduction in plant height and leaf area due to the decrease in cell elongation under drought stress was observed by Shahin et al. (2018b). Drought-linked decline in chlorophyll contents was due to greater activity of chlorophyllase enzyme or destruction of thylakoid membrane; as a result, photosynthetic performance in the form of quantum yield is also reduced (Shen et al., 2020). Drought-tolerant wheat cultivars in this experiment have higher leaf chlorophyll content and quantum yield of PSII than sensitive genotypes (**Figures 3G, H**) producing high yield under drought stress, indicating a positive correlation between chlorophyll content, quantum yield of PSII, and crop yield, in accordance with findings of Chowdhury et al. (2021) and Bano et al. (2021) that drought-tolerant wheat cultivars have greater chlorophyll content and quantum yield.

Wasaya et al. (2021) noted a reduction in spike length, number of grains per spike, and grain weight under drought stress in wheat cultivars, while a reduction in the number of tillers per plant, number of spikelet per spike, and number of grains per spike was observed by Panhwar et al. (2021). The results obtained from the present research suggest that Barani-83, Blue Silver, Pak-81, and Pasban-90 showed better response towards drought conditions, while FSD-08, Lasani-08, Punjab-96, and Sahar-06 cultivars showed poor performance in terms of yield attributes such as the decline in grain yield and 100 grain weight under water shortage is due to reduced production of photo-assimilates and their reduced absorption by sink while the reduction in the number of grains per spike was due to loss of pollen viability as explained in earlier studies by Chowdhury et al. (2021).

Plant water status is measured in terms of RWC, which reflects metabolic activities of plant tissues (Chowdhury et al., 2021). A reduction in RWC was found in all wheat cultivars under drought stress; less reduction in tolerant cultivars (Barani-83 and Blue Silver) while more in sensitive cultivars (FSD-08 and Lasani-08) were found in the present study. These results are in agreement with the previous studies by Belay et al. (2021) who found less reduction in RWC in drought-tolerant wheat cultivars under water-deficit conditions. The decrease in RWC might be due to reduced water uptake and greater loss of water from leaves, which reduces leaf turgor potential, inhibits cell elongation and cell division, and ultimately reduces growth and yield (Camaille et al., 2021). Under such conditions, plants modify their osmotic potential by lowering the water potential in a process called osmotic adjustment, which is carried out by accumulation of suitable solutes like soluble sugars, proline, free amino acids, soluble proteins, and glycine-betaine that increase the tolerance to all types of environmental stresses (Ashraf and Foolad, 2007). The conservation of desirable cellular turgidity under water scarcity permits the plant to keep its physio-metabolic functions such as opening of stomata, photosynthesis, cell expansion, and other developmental processes at optimum level (Serraj and Sinclair, 2002). Cultivars recognized as drought tolerant during the present study maintained water status by accumulation of total soluble sugars, proline, and amino acids (**Figures 6A, B, D**). Different studies have already explained that accumulation of osmo-protectants like soluble sugars, soluble proteins, and proline in leaves is involved in maintaining plant water status under stress conditions (Gontia-Mishra et al., 2016; Blum, 2017; Qayyum et al., 2021). Increase in proline production under drought stress is needed to supply energy for growth and survival to tolerate plants against drought stress (Shahin et al., 2018b). A greater concentration of proline plays an important role in osmotic adjustment, ROS scavenging, and stabilizing cellular components (Hussain et al., 2021). In wheat, the soluble sugars play the most important role in osmotic adjustment during drought stress (Camaille et al., 2021).

Drought-tolerant cultivars Barani-83, Blue Silver, Pak-81, and Pasban-90 showed greater amounts of N, P, and K^+^ accumulated in the leaves as compared to FSD-08, Lasani-08, Punjab-96, and Sahar-06 cultivars under drought stress; previously, it was observed that plants exposed to drought stress have low N content in comparison to well-watered plants as found for rice and wheat due to restricted movement of N and P in the soil and less absorption by roots (Sun et al., 2012). Severe drought reduces N and P contents in leaves of wheat, canola, and soybean plants (Ashraf and Foolad, 2013). Noman et al. (2018) observed reduced levels of K, P, and Ca^2+^ in roots and shoots of wheat plants under drought stress. Oxidative stress refers to generation of ROS in response to drought stress due to shifting of Calvin cycle to photorespiration, which causes lipid peroxidation, chlorophyll etiolation, protein oxidation, and damage to cellular organelles, DNA, and RNA (Abedi and Pakniyat, 2010). Our experimental results are also in accordance with Outoukarte et al. (2019), who reported that enhanced lipid peroxidation and more ROS result in loss of membranes ability to control the rate of ion movement into and out of cells, an indication of plasma membrane damage as revealed by increased MDA content, osmotic stress, and reduced N, P, and K status of drought-sensitive wheat cultivars (FSD-08, Lasani-08, Punjab-96, and Sahar-06) that ultimately affect their final yield.

The effects of drought stress on the shape of raw OJIP curves (Ft) show that Fo and Fj under drought stress were significantly increased, while Fi and Fm were markedly reduced in cvs. FSD-08 and Lasani-08 (**Figures 8A-D**). However, all these fluorescence attributes either remained unchanged or slightly changed in cvs. Blue Silver and Barani-83 due to water stress, which suggests only minor structural damage to PSII at both donor and acceptor end of PSII in these cultivars and can be considered as drought tolerant. Sensitive cvs. FSD-08 and Lasani-08 exhibited increased Fo and decreased energy trapping capacity of PSII, while decreased Fm may be attributed to inactive reaction centers. Damage to oxygen-evolving complex (OEC) reducing Fv values and increasing Vj is an indication of lowering PSII ability to reduce plastoquinone in these cultivars. Similar findings in mung bean were observed by Bano et al. (2021). L-band expresses uncoupling of OEC; its increased value in FSD-08, Lasani-08, and Blue Silver indicated uncoupling of OEC with core protein of PSII, while in Barani-83, no such L-band was observed (**Figure 9A**). Negative K-band values for Blue Silver and Barani-83 show that plants maintain antenna complex and PSII reaction center under the influence of drought stress (**Figure 9B**), while its positive values for cvs. FSD-08 and Lasani-08 show reduced OEC performance due to disturbance in electron flow from OEC to PSII reaction center in accordance with the previous studies, which explain that K-step gives information about the destruction of OEC (Guo et al., 2020). Many studies explain that double normalized differential chlorophyll *a* fluorescence kinetics such as L- and K-bands indicate drought tolerance potential in plants (Brestic and Zivcak, 2013; Kalaji et al., 2018; Zhou et al., 2019). Drought stress caused minor changes in Fv/Fm in cvs. Blue Silver and Barani-83, which suggest no photo-inhibition, while the decrease in PSII efficiency of cvs. FSD-08 and Lasani-08 was mainly due to photoinhibition with greater decrease in Fv/Fm. The results are supported by the findings of Flexas et al. (2009) and Lawlor and Tezara (2009). The quantum yields of primary photochemistry (ΦPo) and the electron transfer from QA to PQ (ΦEo) are slightly affected in drought-tolerant cultivars (Blue Silver and Barani-83), while a greater decrease was observed in cvs. FSD-08 and Lasani-08. However, quantum yield for heat dissipation (ΦDo) increased only in drought-sensitive cultivars FSD-08 and Lasani-08. Fv/Fm and PI_ABS_ are important screening tools to investigate photosynthetic efficiency and effects of abiotic stresses on PSII in different plants. PI_ABS_ depends upon the concentration of active reaction centers of chlorophyll, efficiency of energy trapping, and electron transport from PSII to the plastoquinone pool (Botyanszka et al., 2020). Water stress increased the energy fluxes of absorption (ABS/RC), trapping (TRo/RC), and electron transport (ETo/RC) in FSD-08 and Lasani-08, while it decreased those in Blue Silver and Barani-83 cultivars. Strasser et al. (2000) explained that effective antenna size and absorbed energy are reduced in drought-tolerant cultivars, whereas drought-sensitive cultivars were unable to regulate the antenna size, so active reaction centers of PSII are damaged due to over-excitation. The absorbed energy by antenna proteins is dissipated as heat (DIo/RC) (**Figure 10**).

## 5 Conclusions

A great genetic variability was found among wheat cultivars in the present study. Based on maintaining growth, photosynthetic pigments, and RWC and good osmotic adjustment by accumulating proline, total soluble sugars, and proteins under water stress, Barani-83 and Blue silver were considered the most drought tolerant, while based on disruption of photosynthetic pigments and poor management of water status, FSD-08 and Lasani-08 were ranked as drought-sensitive cultivars. Chlorophyll *a* fluorescence transients confirmed better efficiency of photosystem II in tolerant cultivars (Barani-83 and Blue Silver) due to greater dissipation (DIo/RC) of absorbed energy (ABS/RC) and downregulation of electron transport per reaction center (ETo/RC) to prevent photosystem-II from photooxidative damage. Greater PSII stability and functional activity in drought-tolerant cultivars (Barani-83 and Blue Silver) was evident with greater performance index (PI_ABS_) and greater quantum yield (Fv/Fm) of PSII.

## Data availability statement

The original contributions presented in the study are included in the article/**Supplementary Material**. Further inquiries can be directed to the corresponding authors.

## Author contributions

Conceptualization and designing the experiments, H-u-RA, RA, ZUZ, and SS. Execution of experiments, NH, AG, ZUZ, HB, AK, and MY. Data analysis, NH, MJ, RA, MI, SN, SS, and MZ. Data validation, H-u-RA. First draft writing, NH, AG, and KS. Supervision of the experiment, H-u-RA and ZUZ. Review and editing of the manuscript, H-u-RA, RA, SS, MJ, MI, AK, HB, and MZ. Proofing of the manuscript, H-u-RA, SN, SS, and RA. Providing resources and funding acquisition, RA and SS. All authors contributed to the article and approved the submitted version.
