# Supplementary figures and images for: Photosynthetic activity and metabolic profiling of bread wheat cultivars contrasting in drought tolerance
Source: Front Plant Sci. 2023 Feb 2;14:1123080. doi: 10.3389/fpls.2023.1123080 (PMC9945586; doi:10.3389/fpls.2023.1123080)

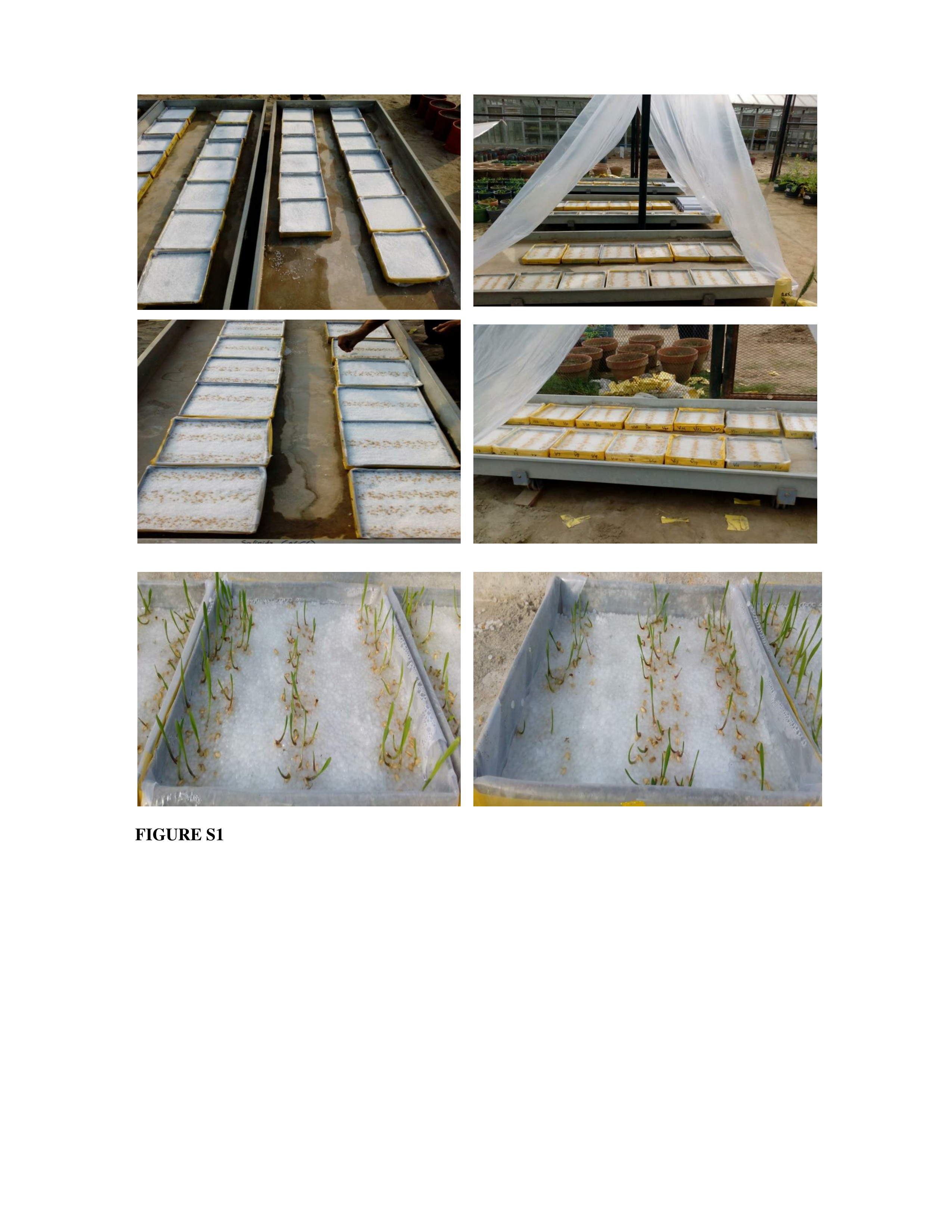

Supplement: Supplementary file 1 [file Image_1.tif]

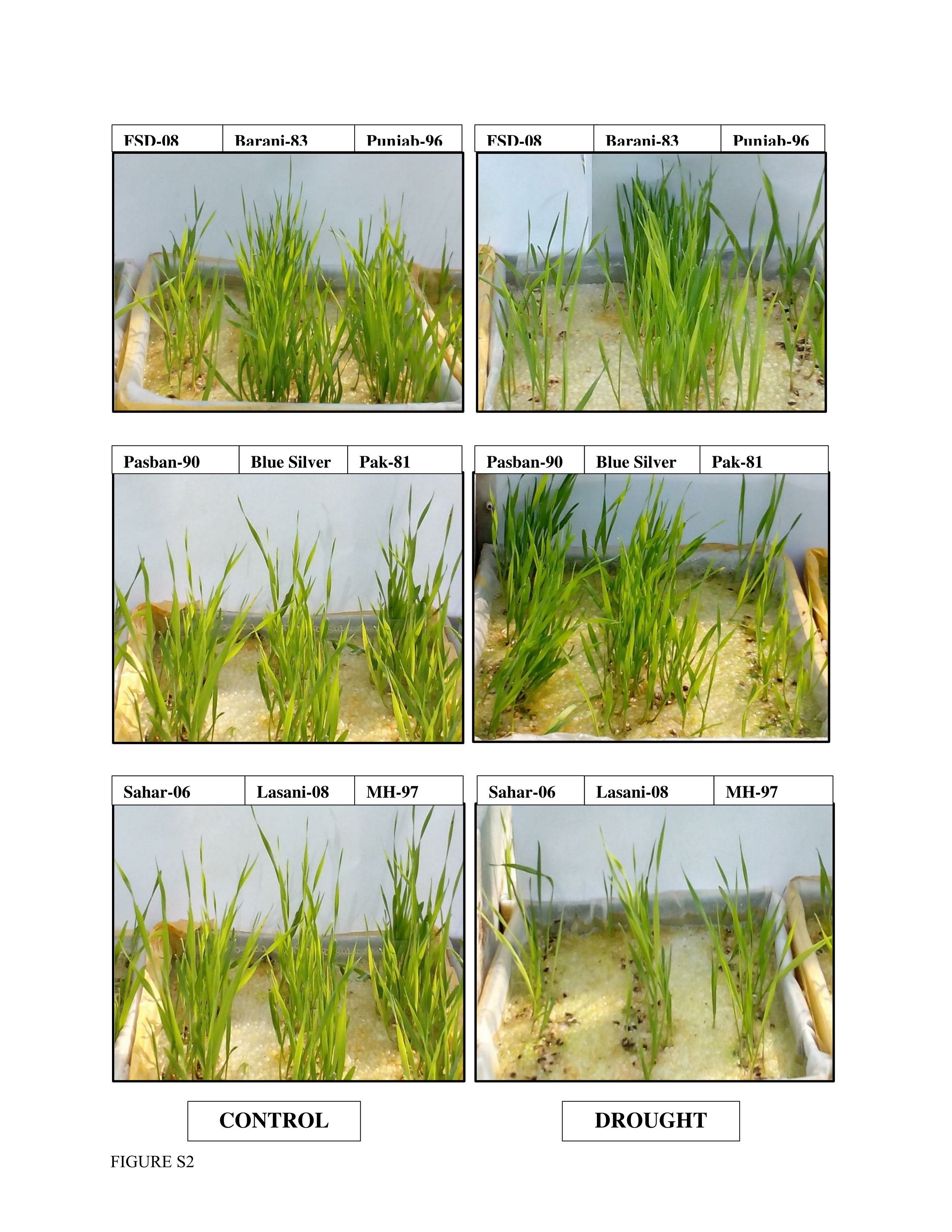

Supplement: Supplementary file 2 [file Image_2.tif]
